# Ultralight, Elastic, Hybrid Aerogel for Flexible/Wearable Piezoresistive Sensor and Solid–Solid/Gas–Solid Coupled Triboelectric Nanogenerator

**DOI:** 10.1002/advs.202204519

**Published:** 2022-10-17

**Authors:** Tianci Huang, Yong Long, Zilong Dong, Qilin Hua, Jianan Niu, Xinhuan Dai, Jiangwen Wang, Junfeng Xiao, Junyi Zhai, Weiguo Hu

**Affiliations:** ^1^ Center on Nanoenergy Research School of Physical Science and Technology Guangxi University Nanning 530004 China; ^2^ CAS Center for Excellence in Nanoscience Beijing Key Laboratory of Micro‐nano Energy and Sensor Beijing Institute of Nanoenergy and Nanosystems Chinese Academy of Sciences Beijing 101400 China; ^3^ School of Nanoscience and Technology University of Chinese Academy of Sciences Beijing 100049 China; ^4^ School of Electronic Communication Technology Shenzhen Institute of Information Technology Shenzhen 518172 China

**Keywords:** aerogel, multifunction, piezoresistive sensors, triboelectric nanogenerators

## Abstract

Aerogels have been attracting wide attentions in flexible/wearable electronics because of their light weight, excellent flexibility, and electrical conductivity. However, multifunctional aerogel‐based flexible/wearable electronics for human physiological/motion monitoring, and energy harvest/supply for mobile electronics, have been seldom reported yet. In this study, a kind of hybrid aerogel (GO/CNT HA) based on graphene oxide (GO) and carboxylated multiwalled carbon nanotubes (CMWCNTs) is prepared which can not only used as piezoresistive sensors for human motion and physiological signal detections, but also as high performance triboelectric nanogenerator (TENG) coupled with both solid–solid and gas–solid contact electrifications (CE). The repeatedly loading–unloading tests with 20 000 cycles exhibit its high and ultrastable piezoresistive sensor performances. Moreover, when the obtained aerogel is used as the electrode of a TENG, high electric output performance is produced due to the synergistic effect of solid–solid, and gas–solid interface CEs (3D electrification: solid–solid interface CE between the two solid electrification layers; gas–solid interface CE between the inner surface of GO/CNT HA and the air filled in the aerogel pores). This kind of aerogel promises good applications for human physiological/motion monitoring and energy harvest/supply in flexible/wearable electronics such as piezoresistive sensors and flexible TENG.

## Graphical abstract

A kind of conductive, ultralight, elastic, hybrid aerogel is prepared by simply physical crosslinking of graphene oxide nanosheets and carboxylated multiwalled carbon nanotubes (CMWCNTs). Piezoresistive sensors for multimode physiological/human motion signal detections with long‐term ultrastability are successfully achieved due to the 3D network structure. High‐performance triboelectric nanogenerator (TENG) is based on the coupling of solid–solid and gas–solid contact electrifications.

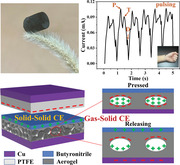

## 1 Introduction

In recent years, great efforts have been made to develop flexible/wearable electronics for physiological signal detections, and energy harvesting. Due to the merits of portability, high energy conversion/harvesting efficiency, flexibility, and stability, flexible/wearable electronics have been widely reported in a wide range of applications, such as piezoresistive sensors for health monitoring, and human motion detections in human–machine interaction, and triboelectric nanogenerators for harvesting tiny mechanical energy. However, most reported flexible electronics, based on a single working mechanism, are usually functionally monotonous. These monofunctional electronics are usually able to sense only one motion or physiological signal,^[^ ^1^, ^2^ ^]^ which limited the practical application ranges. In order to extend the practical applications of flexible/wearable electronics to adapt comprehensive monitoring requirements, it is indispensable to explore wearable electronics with multiple functions to measure and analyze comprehensive physical conditions.^[^ ^2^, ^3^, ^4^, ^5^, ^6^ ^]^ As the active materials for flexible/wearable electronics, excellent electrical conductivity, good flexibility, and reliable long‐term stability are always crucial issues to ensure an efficient and stable working process.^[^ ^7^, ^8^, ^9^ ^]^ Although excellent research works on flexible/wearable electronic devices have been reported recently, and most of them were achieved by directly doping an appropriate amount of conductive components (such as nano metal particles, conductive nanowires, carbon nanotubes, graphene nanosheets) into commercial soft flexible substrates.^[^ ^10^, ^11^, ^12^, ^13^, ^14^ ^]^ However, the functional diversity of these flexible electronic devices is still not enough to adapt to the complex actual environment, for example, real‐time signals for monitoring different types and ranges of human motion (e.g., vocal cord vibrations, facial fault changes, slide frown, joint movements, etc.). Besides, external power sources were always needed to maintain these electronics working, which require additional costs. To address these challenges, triboelectric nanogenerators (TENGs), based on the coupling effects of the triboelectric effect and electrostatic induction, have emerged as a compelling technology to realize the human physiological signal monitoring, human motion detection, and also power these electronics. The ideal electrode materials to meet these requirements should be lightweight, flexible, mechanical robust, and easy to regulate its electrical conductivity.

Aerogels, among the numerous reported materials for flexible/wearable electronics, is the lightest solid in the world. The unique 3D porous structures of aerogels provide high porosity, ultralow density, and high specific surface area.^[^ ^15^, ^16^, ^17^ ^]^ Conductive, compressible, and elastic aerogels have been considered as excellent candidates for fabricating flexible/wearable devices^[^ ^18^, ^19^, ^20^, ^21^ ^]^ such as piezoresistive sensors,^[^ ^15^, ^22^, ^23^, ^24^, ^25^, ^26^, ^27^, ^28^, ^29^ ^]^ and energy harvesting devices.^[^ ^30^, ^31^, ^32^, ^33^, ^34^, ^35^, ^36^, ^37^, ^38^ ^]^ The flexible and compress 3D porous structure of aerogels can provide large numbers of adjustable contact areas which can modulate the electrical resistance under the external pressure. At the same time, compared with traditional TENGs, aerogel‐TENG generate charges from 2D to 3D.

In this work, we prepared a kind of hybrid aerogel (GO/CNT HA) composed of graphene oxide nanosheets (GO) and carboxylated multiwalled carbon nanotubes (CMWCNTs). It was demonstrated that when the obtained GO/CNT HA was used as a piezoresistive sensor, the real‐time signal detection of human physiological/motion was successfully achieved. More importantly, long‐term ultrastability of the piezoresistive sensor was demonstrated by 20 000 cycles’ repetitions of alternatively loading–unloading tests. Additionally, high electrical output performances of the flexible TENG were realized by the GO/CNT HA as the TENG's electrode (GO/CNT HA‐TENG). The enhancement of the GO/CNT HA‐TENG were proved to be due to the coupling of solid–solid CE between the two electrification layers, and gas–solid CE between the 3D framework of the inner GO/CNT HA and the filled air (3D electrification).

## 2 Results and Discussions

### 2.1 Preparation, Mechanical Properties, and Basic Characterization of the GO/CNT HA

**Figure 1:**
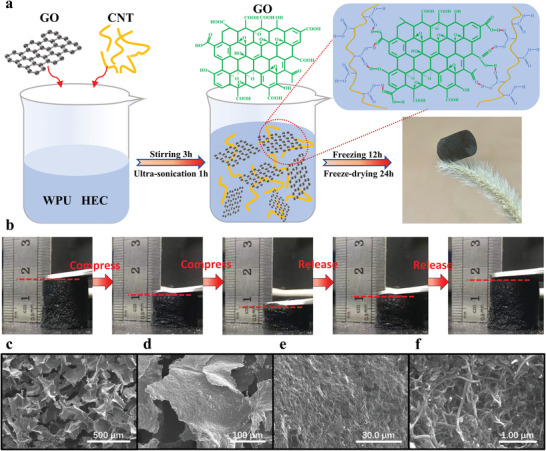
Preparation process, mechanical compress properties, and structure characterization. a) The preparation process of GO/CNT HA. b) Photographs of GO/CNT HA compress and recovery tests. c–f) Scanning electron microscopic images (SEM) of GO/CNT HA at different magnifications.

As shown in **Figure** **1**a, a certain amount of GO dispersion and CNT dispersion were added to the mixed solution of waterborne polyurethane (WPU) and hydroxyethyl cellulose (HEC), and then two steps followed: First, the above mixed solution was treated by magnetic stirring at room temperature and ultrasonic dispersion to form a homogeneous mixture suspension. The HEC showed good dispersibility and adhesion characteristics, which helped to form hydrogen bonds between GO and CMWCNTs, and promoted the solution become gelatinous (precursor of the gel) . In additional, WPU is a green, nontoxic, and excellent elastomer, and the introduction of WPU aqueous suspension as one of the cross‐linkers can assist the formation of the GO/CNT HA aerogels. Second, freeze‐drying method was used to remove the free water from the gel. GO nanosheets contain a large number of oxygen‐containing functional groups, such as hydroxyl, carboxyl, and epoxy groups. CMWCNTs have good conductivity and contain a large number of carboxyl groups. During the formation of aerogel, hydrogen bonds were formed between GO nanosheets and CMWCNTs. At the same time, 2D GO nanosheets and 1D CMWCNTs are physically connected and interwoven with each other, eventually forming a stable 3D porous conductive framework structure. The resultant aerogel showed so lightweight that even a green bristlegrass can easily support it without any collapse of the bristlegrass itself. The mechanical properties of compress tests of GO/CNT HA are shown in Figure 1b and Video S1 (Supporting Information). The GO/CNT HA can still recover to the original state at a compress strain of 65%, proving its good mechanical properties. The internal 3D network structure of GO/CNT HA was characterized by scanning electron microscope (SEM), from which we can observe the uniform porous framework structure, and the CMWCNTs are uniformly attached to the GO nanosheets (Figure 1c–f). In order to characterize the pore size distribution and the specific surface area of the aerogel more accurately, BJH (Barrett–Joyner–Halenda) method, and BET (Brunauer–Emmett–Teller) method from N_2_ gas adsorption isotherms were performed. The average pore size was calculated to be 59.4 nm and the specific surface area to be 1.46 m^2^ g^−1^.

### 2.2 The Mechanical/Electrical Characteristics of GO/CNT HA

**Figure 2:**
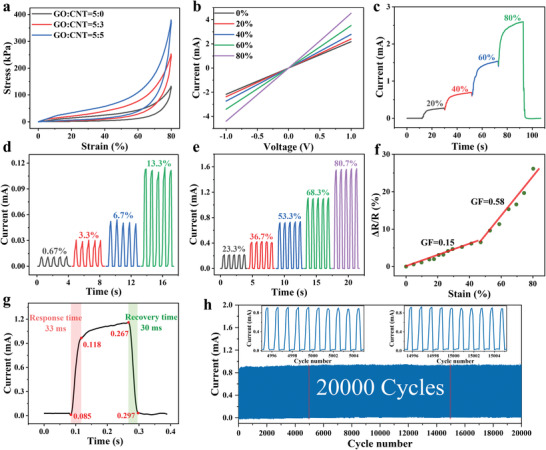
The mechanical/electrical characteristics of GO/CNT HA. a) Stress–strain curve of GO/CNT HA with different doping amount of CMWCNTs (strain from 0% to 80%). b) *I–V* curves of GO/CNT HA under different compress strain. c) *I–t* curves of GO/CNT HA under different compress strain. d) *I–t* curves of GO/CNT HA under loading–unloading with low compress strain. e) *I–t* curves of GO/CNT HA under loading–unloading with high compress strain. f) The gauge factor of GO/CNT HA within the compress strain range of 0–80%. g) The response and recovery time of GO/CNT HA at 65% compress strain. h) *I–t* curves under repeatedly loading‐unloading tests of GO/CNT HA with 20 000 cycles at 60% compress strain.

In order to explore the mechanical properties of GO/CNT HA, compress stress–strain experiments were carried out. To investigate the influences of the doped CMWCNTs on the mechanical properties of GO/CNT HA, aerogels with different amounts of CMWCNTs were prepared by the same preparing method. The corresponding compress stress‐strain curves of aerogels with different amounts of CMWCNTs were shown in **Figure** **2**a, from which we can observe that with the increase of the amount of the doped CMWCNTs, the corresponding GO/CNT HA can withstand higher compress stress under the same compress strain. In the compress process, the compress stress–strain curve can be divided into two stages (the linear stage: the compress strain less than 50%, the nonlinear stage: 50–80%). During the linear stage, as the air filled in the aerogel pores were gradually extruded, the pores in the GO/CNT HA were compressed. The main changes that took place during this stage were the deformations of the pores. However, in the nonlinear stage, as the deformation became larger and larger, the inner surfaces of the GO/CNT HA gradually approach and squeeze each other. Consequently, the compress stress increased significantly at this stage. Because CMWCNTs have good conductivity. In theory, the more the CMWCNTs doped, the better the conductivity of the obtained GO/CNT HA. However, on the other hand, the more CMWCNTs doped, the greater the weight of the GO/CNT HA. To balance the relationship between the weight and conductivity of GO/CNT HA, GO/CNT HA with the same CNT doping content as GO (GO:CNT = 5:5) was adopted.

To evaluate the piezoresistive sensor performance of the GO/CNT HA, the current–voltage curves (*I–V* curves) under different compress strains were tested, respectively, and the results showed a good linear relationship, as shown in Figure 2b. The slopes of the *I–V* curves increased with the increase of the compress strain, the reason of which is due to the increase of the conductive contact points inside the GO/CNT HA. When external pressure is applied on the GO/CNT HA, excellent piezoresistive sensory properties were produced. Specifically, when the 3D macroscopical GO/CNT HA is compressed under external pressure, more GO nanosheets/CNT in the inner surfaces of the aerogel moved closer to each other, making the internal conductive contact points increase. The working mechanism of aerogel during compression is shown in Figure S1 (Supporting Information). Consequently, the resistance of GO/CNT HA decreased. On the contrary, as the external pressure gradually removed, the compressed network structure of the GO/CNT HA gradually restored to its original state. As the GO/CNT HA are conductive and have good mechanical properties, when the GO/CNT HA was connected to a circuit with a small LED bulb in series under a given voltage of 3 V, as shown in Video S2 (Supporting Information), the brightness of LED changed with the compress strain of the aerogel varied because of the resistance changing when the aerogels are compressed. When the external pressure was removed, the GO/CNT HA recovery to its original state, and the resistance also returned to its original value, and the LED became darker. Consequently, this kind of hybrid structure made the GO/CNT HA suitable for piezoresistive sensor applications. As shown in Figure 2c, the current–time curve (*I–t* curve) of GO/CNT HA, the current increases significantly with the increase of the corresponding compression strain (20%, 40%, 60%, 80%). The contact regions of the inner surfaces of GO/CNT HA greatly increased, resulting in a greater increase in current and a steady current signal. Under different compress strains, the *I–t* curves of the compress–release were tested, as shown in Figure 2d,e. The results showed that even under a compress strain as low as 0.67%, or as high as 80.7%, the corresponding peak of the cyclic compress–release repeated steadily, indicating the excellent elasticity of the GO/CNT HA and it can restore to its original state quickly after compressed. In order to quantitatively evaluate the sensing performance of GO/CNT HA, the gauge factor (GF) of the piezoresistive sensor is calculated by the following formula

(1) $$\mathrm{GF}\;=\left(\Delta R/R_{0}\right)/\varepsilon \;=\left(\frac{R_{0}-R}{R_{0}}\right)\;/\varepsilon$$

Where the “*ε*”, “*R*”, and “*R*_0_” respectively the compress strain, resistance during compression and resistance at initial stage of GO/CNT HA. As shown in Figure 2f, the GF was divided into two sections, GF = 0.15 at 0–50% compress strain and GF = 0.58 at 50–80% compress strain. The response time and recovery time of GO/CNT HA under compress strain of 65% can reach as fast as 33 and 30 ms, respectively, as shown in Figure 2g, which were due to the three‐dimensional conductive network structure of the GO/CNT HA worked as springs. The compression response of aerogel is quite sensitive when it is under pressure, and it recovers immediately after the pressure is eliminated. To assess the stability and durability of GO/CNT HA in piezoresistive sensor application, 20 000 repetitions of continuous cyclic compress tests were carried out under 60% compress strain. As shown in Figure 2h, each enlargement of two random parts showed no obvious fluctuation, and is consistent with the initial current signal, indicating that the GO/CNT HA has excellent structural and sensor stability.

### 2.3 GO/CNT HA Piezoresistive Sensor to Monitor Human Signals

**Figure 3:**
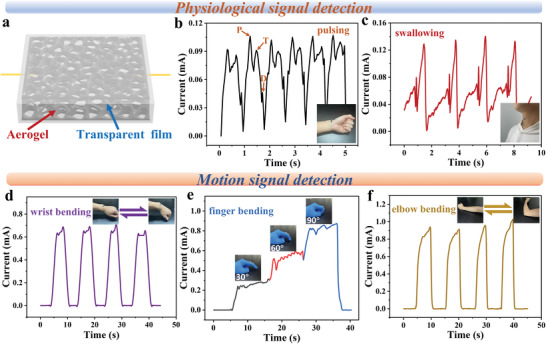
GO/CNT HA piezoresistive sensor to monitor human signals. Physiological signal detection: a) Structure diagram of GO/CNT HA piezoresistive sensor. b) The signals of pulsing wave and waveform consisting of three characteristic peaks. c) The signal of swallowing; Motion signal detection: d) The signal of wrist repeatedly bending. e) The signal of finger bending at different angles. f) The signal of elbow repeatedly bending.

As were discussed above, the GO/CNT HA has been demonstrated good conductivity, sensitive piezoresistive response, and excellent structural and sensor stability. Therefore, applications of piezoresistive sensors for various kinds of human signal detections were performed by simply encapsulating the GO/CNT HA and adhering to human skin. Due to the different motion types and moving range of the human body, the detected human signals could be classified into “ Physiological signal” and “Motion signal.” On one hand, with respects to “ Physiological signal” monitoring: The GO/CNT HA was cut into small squares pieces with the thickness of 2 mm, transparent film covered the GO/CNT HA slices to protect it from damage (**Figure** **3**a). Based on fast response and high stability, the GO/CNT HA piezoresistive sensor can clearly detect and record the real‐time pulsing signal of the human body by been attached to the wrist pulse position. As shown in Figure 3b, the three characteristic peaks relating to percussion wave (P), tidal wave (T), and diastolic wave (D) can be clearly identified from a single pulse waveform, which may provide useful information for the diagnosis of cardiovascular diseases. Besides, the GO/CNT HA piezoresistive sensor can monitor the swallowing movement in real‐time by adhering the aerogel to the throat (Figure 3c). On the other hand, for the situation of “Motion signal” monitoring: When the GO/CNT HA piezoresistive sensor is attached to the wrist, the real‐time wrist activity signals could be detected. As shown in Figure 3d, the wrist bent and stayed for a short time, and then returned to the original state and repeated the same action many times, the corresponding real‐time current signals were all clearly recognized. When the GO/CNT HA piezoresistive sensor is connected to the joints of fingers, the activity signals of fingers at different angles could be recorded (Figure 3e). With the increase of finger bending angle, the resistance of aerogel compressing decreased and the current value of sensor increased. When the GO/CNT HA piezoresistive sensor was attached to the elbow, as shown in Figure 3f, movements of elbow extension signals (bending and stretching) could be detected. In addition, when writing on the surface of the transparent film on the top of the GO/CNT HA piezoresistive sensor, the GO/CNT HA under the touch point of the nib was compressed, and the resistance of the aerogel immediately changed, resulting in a real‐time change in the output current (Figure S2, Supporting Information). When writing different letters such as “G”, “X”, and “U” on the transparent film, respectively, the piezoresistive sensor produced a unique real‐time current signal due to the different stroke and intensity of the different letter. For the same letter, the corresponding real‐time piezoresistive current signal could be reproduced stably, indicating that the GO/CNT HA piezoresistive sensor can identify individual writing habits. To further study the piezoresistive sensor performances of the GO/CNT HA, a 4×4 sensor array was assembled with sixteen cylindrical pieces of GO/CNT HA. For example, we randomly pressed two sensor units of the 4×4 sensor array at the same time, as shown in Figure S3a (Supporting Information), and the corresponding real‐time voltage signals were simultaneously collected and displayed in the form of three‐dimensional bar distribution (Figure S3b). GO/CNT HA piezoresistive sensor has excellent performance.

### 2.4 Construction of Solid–Solid/ Gas–Solid Coupled TENG

**Figure 4:**
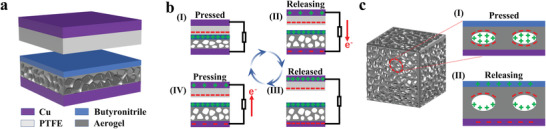
Structure and mechanism of the GO/CNT HA‐TENG. a) Schematic of the layer‐by‐layer GO/CNT HA‐TENG structure. b) Working mechanism of GO/CNT HA‐TENG. c) Working mechanism of gas–solid friction power generation.

As a multifunctional flexible/wearable electronic, the GO/CNT HA also exhibited excellent electric output performances in the applications of triboelectric nanogenerator (TENG), a new kind of energy conversion and harvesting electronics invented by Wang in 2012.^[^ ^39^, ^40^, ^41^, ^42^ ^]^ As we know, for TENGs, there were four basic working modes: vertical contact separation mode, horizontal sliding mode, single electrode mode, and independent layer mode. Here in our study, a typical sandwich TENG structure with vertical contact separation mode was used, and the GO/CNT HA was used as the TENG electrode (the TENG was abbreviated as “GO/CNT HA‐TENG”). The aerogel electrode was covered by a piece of commercial butyronitrile layer as one of the electrification layers, and PTFE film as the opposite electrification layer (**Figure** **4**a). Based on the coupling effect of triboelectrification and electrostatic induction, when two different materials contacted, electrons generated on the two contact surfaces, PTFE obtained electrons due to negative electron affinity, and butyronitrile layer lost electrons because of its relative positive electron affinity, as shown in Figure 4b (I). It is worth noting that when the PTFE layer is in contact with butyronitrile layer, the aerogel electrode is compressed, causing the two types of contacts: the solid–solid interface contact (PTFE layer and butyronitrile layer) and solid–gas contact (aerogel inner surface and air in pores). Solid–gas contact led to the charge transfer from air to the relatively negative aerogel, resulting in the accumulation of negative charges inside the aerogel and positive air charge in the aerogel pores, as shown in Figure 4c (I). Consequently, the coupling effect of solid–solid and solid–gas triboelectrification promoted the final electric output of the GO/CNT HA‐TENG enhanced. When the gap between the PTFE and the butyronitrile layer increased, the deformation of the compressed pores in the aerogel recovered, and the opposite triboelectrification charges were separated, as shown in Figure 4b (II),c (II), and then an electric potential difference generated to drive the induced electrons to flow from the top electrode to the bottom electrode, and the transient current flew continuously through the external circuit until an electrostatic equilibrium is achieved. When the external force is reapplied on the GO/CNT HA‐TENG, the electrons reflow and form a reverse current. The alternative mechanical loading–unloading processes made the electrons flow back and forth in the external circuit.

### 2.5 Electric Output Performances and Practical Applications of the GO/CNT HA‐TENG

**Figure 5:**
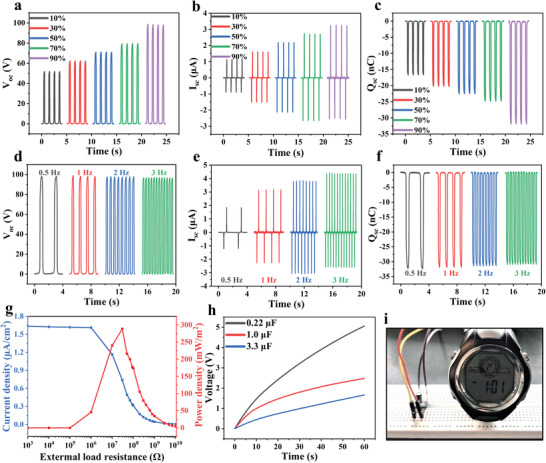
Electric output performances and practical applications of the GO/CNT HA‐TENG. a–c) *V*_OC_, *I*_SC_, and *Q*_SC_ under different compress strain: 10%, 30%, 50%, 70%, and 90% and operating frequency of 1 Hz. d–f) *V*_OC_, *I*_SC,_ and *Q*_SC_ under different operating frequency: 0.5, 1.0, 2.0, and 3.0 Hz and the compress strain of 90%. g) Current density and power density under the different external load resistances. h) The charging curve of the GO/CNT HA‐TENG for different capacitors under a compress strain of 90% at a frequency of 1 Hz. i) The optical image showing that a commercial electronic watch was successfully driven by the GO/CNT HA‐TENG.

To further study the output performance of GO/CNT HA‐TENG, we carried out a series of control experiments. The contact–separation mechanical movements were driven and controlled by a linear motor with the frequency of 1 Hz and the speed of 2 m s^−1^. The contact area could be calculated to be *S* = 1.767cm^2^ (*S* = *πr* ^2^, r = 7.5 mm). The GO/CNT HA‐TENG output performances under different compress strains (10–90%) are measured. The open‐circuit voltages (*V*_OC_) increased from 51.7 to 98.4 V (**Figure** **5**a). The short‐circuit currents (*I*_SC_) increased from 1.01 to 2.89 μA (Figure 5b). The charges transferred under short‐circuit (*Q*_SC_) increased from 16.6 to 31.9 nC (Figure 5c). From the above measured results of *V*_OC_, *I*_SC_, and *Q*_SC_, it can be proved that the greater the compress strain of aerogel electrode, the better the output performances of GO/CNT HA‐TENG. To confirm this conclusion, a set of control experiments were carried out by replacing the aerogel electrode with a piece of ordinary copper foil (Copper‐TENG), and the corresponding *V*_OC_ was measured to be 62.5 V (Figure S4a, Supporting Information), the *I*_SC_ was 2.25 μA (Figure S4b, Supporting Information), the *Q*_SC_ was 19.8 nC (Figure S4c, Supporting Information). By comparison, with copper foil as the electrode, the Copper‐TENG electric output performances were obviously lower than the GO/CNT HA‐TENG electric output performances when the compress strain of the aerogel electrode reached 50–90%. The high electric output performances of GO/CNT HA‐TENG under large compress strain is attributed to solid–solid and gas–solid friction coupling power generation and the reduction of GO/CNT HA resistance. Compared with traditional TENGs, GO/CNT HA‐TENG generate charges from 2D to 3D and 3D electrification is the unique advantage of GO/CNT HA‐TENG.

In addition to the factors of different compress strain discussed above, in the natural environment, the mechanical motion was always random, and its amplitude and frequency were always irregular. Therefore, the mechanical motion frequency in the working process of GO/CNT HA‐TENG was another key factor that may affect the energy collection efficiency of GO/CNT HA‐TENG. Consequently, in our experiments, the frequencies of the external mechanical force were increased from 0.5 to 3 Hz. As shown in Figure 5d,f, at the frequencies of 0.5, 1.0, 2.0, 3.0 Hz, the measured *V*_OC_ showed no obvious change, and so did the *Q*_SC_. However, the *I*_SC_ increased from 1.52 to 3.48 μA, as shown in Figure 5e, which were consistent with the variation tendency of TENGs. Under the working frequency of 1 Hz, different external load resistances were used to evaluate the power density of the GO/CNT HA‐TENG, as shown in Figure 5g. As the external resistance increased from 1 KΩ to 10 GΩ, the peak output current density decreased. When the external resistance was up to 30 MΩ, the corresponding current density was 0.737 μA cm^−2^, and the maximum instantaneous power density (*P*) was calculated to be 288 mW m^−2^ ($P\;=\frac{I^{2}R}{S}\;$, Where “R” represents the load resistance, “*I*” represents the output peak current through the external load, and “*S*” represents the contact area between PTFE and butyronitrile film). Compared with the state‐of‐the‐art in the literature reports, the power density of GO/CNT HA‐TENG is relatively high (as shown in Table S1 in the Supporting Information).^[^ ^38^, ^43^, ^44^, ^45^, ^46^, ^47^, ^48^, ^49^, ^50^ ^]^ Finally, the GO/CNT HA‐TENG was used to charge commercial capacitors, as shown in Figure 5h. When the working frequency is 1 Hz, three different capacitors (0.22, 1.0, and 3.3 µF) could be charged for 60 s through the rectifier, and charge the voltage of the capacitor to 5.05, 2.47, and 1.66 V respectively. By charging commercial capacitors, small portable electronic devices (watch) can be directly driven, as shown in Figure 5i and Video S3 (Supporting Information).

## 3 Conclusion

In summary, a kind of conductive aerogel (GO/CNT HA) with 3D network structure was prepared by freeze‐drying method. The aerogel showed excellent mechanical and electrical properties (flexibility, elasticity, compressibility, and conductivity). On this basis, multifunctional flexible/wearable electronics were constructed, including piezoresistive sensors for various human signal detections (physiological signal and motion signal), and triboelectric nanogenerator for mechanical energy harvest. The excellent mechanical properties and long‐term stability of the aerogel was due to its unique physical structure (2D GO nanosheets and 1D CMWCNTs physically interconnected and intertwined with each other) and chemical structure (hydrogen bonds formed between GO nanosheets and the CMWCNTs). Piezoresistive sensor with the advantage of detecting different types and ranges of human motion is due to the good elasticity, compressibility, and the 3D porous framework structure, which resulting the contact/separation between the conductive inner surfaces with the external pressure alternatively loading–unloading. The high enhanced electric output performances of the GO/CNT HA‐TENG were attribute to the coupling effect of solid–solid and gas–solid triboelectrification (3D electrification). The obtained GO/CNT composite aerogel in this work, to a certain extent, overcame the bottleneck of single function of flexible wearable electronic devices, and realized multi‐functional applications.

## 4 Experimental Section

### Materials

Water polyurethane (WPU, solid content: 40%) was purchased from Shanghai McLean Biochemical Co., Ltd. hydroxyethyl cellulose (HEC, 80–125 mPa s, 25 °C) was purchased from Aladdin. carboxylated multiwalled carbon nanotubes aqueous dispersion (CMWCNTs, carbon nanotube content: ≈10 wt%) was purchased from XFNANO. All reagents are analytical reagents.

### Preparation of GO/CNT HA

(1) 3.0 g HEC was added into 100 mL DI water at room temperature and stir until it was completely dissolved. (2) 1.0 g GO nanosheets, prepared according to a modified Hummer method,^[^ ^51^ ^]^ was added into 200 mL DI water, stir at room temperature for 30 min, and then ultrasonic treatment for 30 min until the aqueous suspension became dispersed homogeneously. (3) 4 mL WPU and 10 mL HEC was then added into 6 mL DI water (in 50 mL beaker) and keep stir. (4) Adding 10 mL 5 mg mL^−1^ GO and 10 mL CNT to the beaker. The mixed solution was stirred for 3 h, and then ultrasonic for 1 h (300 W), gel solution was obtained. (5) The gel was introduced into the template, frozen for 12 h (−20 C), and then freeze–dried for 24 h, and then the ultralight GO/CNT HA was obtained.

### Experimental Equipment

Microstructure of GO/CNT HA scanned by SU8020 (Hitachi) cold field scanning electron microscope. YueLian tension machine was used to compress stress–strain test for GO/CNT HA. Shanghai Chen Hua CHI660E electrochemical workstation was used for GO/CNT HA *I–t* curve test. Linear motor (LinMot E1100) was used to provide driving force for TENG contact separation process. The electric output performance (*V*_OC_, *I*_SC_, and *Q*_SC_) of TENG was detected and recorded by Keithley 6514 electrometer.

## Conflict of Interest

The authors declare no conflict of interest.

## Supporting information

Supporting Information

Supplemental Video 1

Supplemental Video 2

Supplemental Video 3

## Data Availability

The data that support the findings of this study are available from the corresponding author upon reasonable request.
