# Supplementary material for: Ultralight, Elastic, Hybrid Aerogel for Flexible/Wearable Piezoresistive Sensor and Solid–Solid/Gas–Solid Coupled Triboelectric Nanogenerator
Source: Adv Sci (Weinh). 2022 Oct 17;9(34):2204519. doi: 10.1002/advs.202204519 (PMC9731684; doi:10.1002/advs.202204519)
Supplement: Supplementary file 1 — Supporting Information [file ADVS-9-2204519-s002.pdf]

Supporting Information

Ultralight, elastic, hybrid aerogel for flexible/ wearable piezoresistive sensor and solid-solid/ gas-solid coupled triboelectric nanogenerator

Tianci Huang, Yong Long, Zilong Dong, Qilin Hua, Jianan Niu, Xinhuan Dai, Jiangwen Wang, Junfeng Xiao*, Junyi Zhai*, Weiguo Hu*

Tianci Huang, Zilong Dong, Junyi Zhai, Weiguo Hu
Center on Nanoenergy Research, School of Physical Science and Technology, Guangxi University, Nanning 530004, China

Tianci Huang, Yong Long, Zilong Dong, Qilin Hua, Jianan Niu, Xinhuan Dai, Jiangwen Wang, Junyi Zhai, Weiguo Hu
CAS Center for Excellence in Nanoscience, Beijing Key Laboratory of Micro-nano Energy and Sensor, Beijing Institute of Nanoenergy and Nanosystems, Chinese Academy of Sciences, Beijing 101400, China

Yong Long, Qilin Hua, Jianan Niu, Xinhuan Dai, Jiangwen Wang, Junyi Zhai, Weiguo Hu
School of Nanoscience and Technology, University of Chinese Academy of Sciences, Beijing 100049, China

Junfeng Xiao, Weiguo Hu
School of Electronic Communication Technology, Shenzhen Institute of Information Technology, Shenzhen 518172, China

Email Address: huweiguo@binn.cas.cn

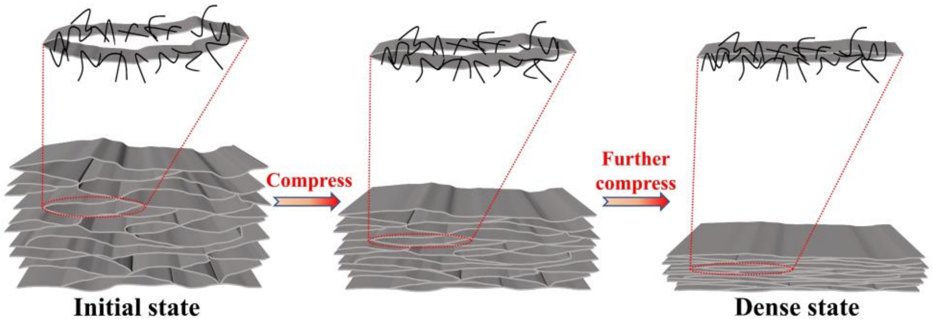

Figure S1. Schematic diagram of structure of aerogel under compression.

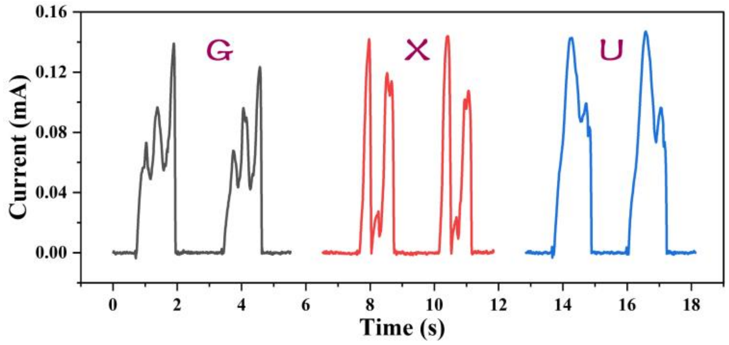

Figure S2. The signals of writing different word.

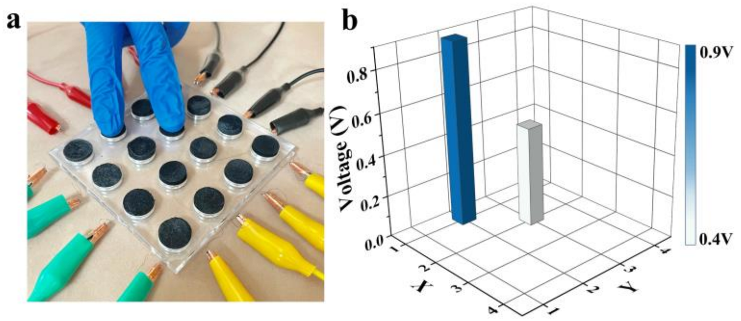

Figure S3. (a) Photograph of the 4x4 sensor array touched by two fingers. (b)Corresponding 3D display diagram.

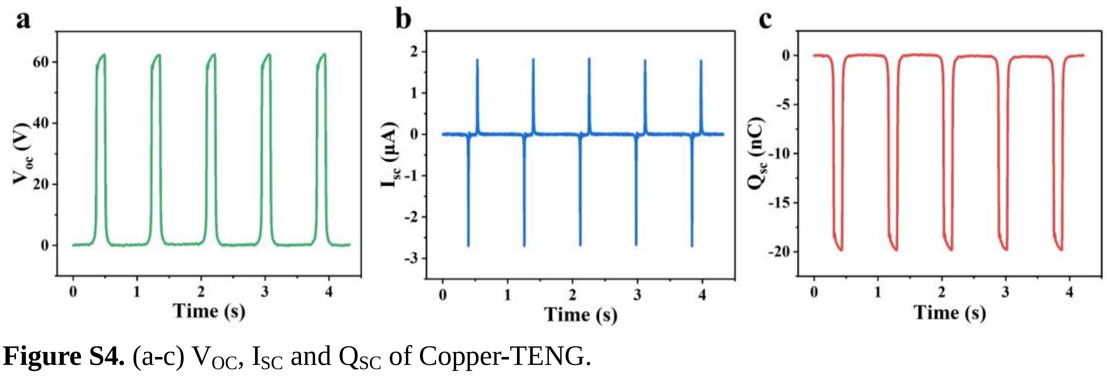

Figure S4. (a-c) V_{OC}, I_{SC} and Q_{SC} of Copper-TENG.

Table S1. compared the power density of TENG with the state-of-the-art in the literature reports.

Triboelectric materials	Power density (mW/m^2)	Ref
**PDMS/liquid crystalline poly**	0.03	[43]
**PTFE/PI/Oil**	1.23	[44]
**md-PI/Al**	1.42	[45]
**PTFE/Human skin**	3.8	[46]
**Cu/Kapton**<br>**Cu/PTFE**	5.17	[47]
**PDMS/PI**	6.2	[48]
**Al/PP fabric**	18	[49]
**Aerogel/PTFE**	127	[38]
**TPU/PDMS**	200	[50]
**PTEF/ butyronitrile**<br>**Aerogel/air**	288	This work

Video 1. Compress recovery properties of GO/CNT HA.

Video 2. The compress recovery of GO/CNT HA control the brightness of LED.

Video 3. GO/CNT HA-TENG driven electronic watch.
